# Polymorphisms in *nucleotide excision repair* genes and susceptibility to colorectal cancer in the Polish population

**DOI:** 10.1007/s11033-014-3824-z

**Published:** 2014-11-13

**Authors:** Katarzyna Paszkowska-Szczur, Rodney J. Scott, Bohdan Górski, Cezary Cybulski, Grzegorz Kurzawski, Dagmara Dymerska, Satish Gupta, Thierry van de Wetering, Bartłomiej Masojć, Aniruddh Kashyap, Paulina Gapska, Tomasz Gromowski, Józef Kładny, Jan Lubiński, Tadeusz Dębniak

**Affiliations:** 1 Department of Genetics and Pathomorphology, International Hereditary Cancer Center, Pomeranian Medical University, Polabska 4, Szczecin, Poland; 2 Discipline of Medical Genetics, Faculty of Health, University of Newcastle and Hunter Medical Research Institute, Newcastle, NSW Australia; 3 Division of Molecular Medicine, Hunter Area Pathology Service, John Hunter Hospital, Newcastle, NSW 2305 Australia; 4 Postgraduate School of Molecular Medicine, Warsaw Medical University, Ul. Księcia Trojdena 2a, 02-091 Warsaw, Poland; 5 Department of General and Oncological Surgery, Pomeranian Medical University, Al. Powstańców Wlkp. 72, 70-111 Szczecin, Poland

**Keywords:** Colorectal cancer risk, NER system, Xeroderma pigmentosum genes, XP genes, XPC, XPD

## Abstract

Xeroderma pigmentosum (XP) is a rare autosomal recessive disease that is associated with a severe deficiency in nucleotide excision repair. Genetic polymorphisms in XP genes may be associated with a change in DNA repair capacity, which could be associated with colorectal cancer development. We assessed the association between 94 single nucleotide polymorphisms (SNPs) within seven XP genes (*XPA*–*XPG*) and the colorectal cancer risk in the Polish population. We genotyped 758 unselected patients with colorectal cancer and 1,841 healthy adults. We found that a significantly decreased risk of colorectal cancer was associated with *XPC* polymorphism rs2228000_CT genotype (OR 0.59; p < 0.0001) and the rs2228000_TT genotype (OR 0.29; p < 0.0001) compared to the reference genotype (CC). And an increased disease risk was associated with the *XPD* SNP, rs1799793_AG genotype (OR 1.44, p = 0.018) and rs1799793_AA genotype (OR 3.31, p < 0.0001) compared to the reference genotype. Haplotype analysis within *XPC*, *XPD* and *XPG* revealed haplotypes associated with an altered colorectal cancer risk. Stratified analysis by gender showed differences between the association of three SNPs: *XPC* rs2228000, *XPD* rs1799793 and *XPD* rs238406 in females and males. Association analysis between age of disease onset and polymorphisms in *XPD* (rs1799793) and *XPC* (rs2228000) revealed differences in the prevalence of these variants in patients under and over 50 years of age. Our results confirmed that polymorphisms in *XPC* and *XPD* may be associated with the risk of colorectal cancer.

## Introduction

Colorectal cancer was second most commonly reported malignancy in 2010 in Poland. It represented 12.4 % of all diagnosed male and 10.1 % female malignancies, respectively [1]. Worldwide colorectal carcinoma was the third most frequently diagnosed cancer in 2009 [2] yet it is preventable if diagnosed early. Colorectal cancer risk is attributable to rare germline and or somatic mutations in a variety of tumor suppressor genes, that include *APC* [3, 4], *TP53* [5]; proto-oncogenes, *KRAS, BRAF, CTNNB1/beta*-*catenin, PIK3CA, SRC* [4]; cell cycle regulatory genes *FBXW7* [4]; DNA mismatch repair genes *MLH1, MSH2, MSH6, PMS2* [6], DNA base excision repair genes *OGG1* [7]*, MUTYH* [8]; and many other common, low-penetrant genetic variants, which together may be associated with colorectal cancer development [9].

Nucleotide excision repair (NER) genes play a crucial role in the maintenance of genomic integrity by removing UV-light-induced DNA lesions [10] as well as those that are a result of UV-mimetic agents. Given the environmental exposure of the colon and rectum to a variety of genotoxic agents any change in the fidelity of this process may influence the risk of disease development. Numerous polymorphisms of NER genes have been identified and individually or in combination may adversely affect NER fidelity, which could contribute to the risk of colorectal cancer. A number of studies have examined whether there is a correlation between NER gene polymorphisms and colorectal cancer risk. The results to date, however, remain inconclusive and do not provide any clear direction concerning their involvement in CRC. Several reports suggest an association between NER genes and colorectal cancer risk [11–16] whereas others have indicated no correlation with disease [17–23].

The aim of this study was to examine associations between genetic variants in DNA repair genes and colorectal cancer risk in the Polish population-based case–control data set. A total of 15 SNPs (selected from a panel of 94 polymorphisms as described previously [24] ) were examined in 758 unselected patients with colorectal cancer and their frequency compared to that found in 1,841 healthy adults. Logistic regression and haplotype analysis was undertaken to assess the impact of these polymorphisms on CRC genetic susceptibility.

## Patients and methods

### Patients

For this study a group of 758 unselected colorectal cancer patients were invited to participate in this research: There were 355 women (mean age at diagnosis, 62.61 years) and 403 men (mean age at diagnosis, 63.34 years) from Poland. The minimum age at onset was 23 years among men and 27 among women and the maximum age at onset was 92 in both male and female. An early-onset CRC (<50) was present in 17.3 % cases (men : 16.6 %; women: 17.7 %). Data on cancer location was available for 625 cases. Of the 625 cases, 118 individuals were diagnosed with right-sided CRC, 467 were diagnosed with left-sided cancer and 40 cases had tumors in an unspecified location of the colon. Tumors from the cecum through the transverse colon were classified as right-sided colon cancers, tumors from the splenic flexure to rectum were considered to be left side colon cancers. Patients were diagnosed between the years 2005 and 2008 at the Surgical Oncology Clinic, Pomeranian Medical University, Szczecin. The registries used to identify patients captures over 95 % of all diagnosed cancers within the geographical region of Szczecin.

The control group consisted of 1,841 healthy adults: 860 women (mean age, 64 years) and 981 men (mean age, 67 years). These healthy adults had a negative cancer family history for first- and second-degree relatives defined by answering a questionnaire about their family’s medical history. This was part of a population-based study of the 1.5 million residents of West Pomerania aimed at identifying familial aggregations of malignancies performed recently by our center. During the interview, the goals of the study were explained, informed consent was obtained, genetic counseling was given and a blood sample taken for DNA analysis. Individuals affected with any malignancy or with cancers diagnosed among first- or second- degree relatives were excluded from the study.

Informed consent was obtained from all participants prior to the collection of a blood sample for DNA isolation. The study was approved by the institutional review board of the Pomeranian Medical University.

### Methods

#### SNPs selection

The 94 SNPs located in the seven DNA repair genes (XPA: 8 SNPs, XPB: 10 SNPs, XPC: 16 SNPs, XPD: 11 SNPs, XPE: 5 SNPs, XPF: 16 SNPs XPG: 28 SNPs) were selected in a previous study from http://snpper.chip.org/ [25] (for details see [24]). All single-nucleotide polymorphisms were non-synonymous or present within the exon/intron boundaries of the respective gene or located in the 5′-UTR and 3′-UTR sequences. The minimum threshold for minor allele genotype frequency was set at 5 %. Out of 94 SNPs present within the 7 genes 15 were eligible for detailed analysis (Table 1).

**Table 1:** List of all examined SNPs

Gene	SNP	Function	Genotype	Cases	Controls	p value	OR (95 % Cl)	HWE
XPC	G2061A	Arg687Ser	GG	229 (59 %)	827 (54.8 %)	–	–	0.27
			AG	135 (35 %)	592 (39.2 %)	0.1126	0.82 (0.65–1.05)	
			AA	24 (6 %)	91 (6 %)	0.7364	0.92 (0.57–1.49)	
	G1475A	Arg492His	GG	415 (90,8 %)	1,197 (91.1 %)	–	–	0.094
			AG	40 (8.8 %)	116 (8.9 %)	0.99	1.00 (0.69–1.50)	
			AA	1 (0.4 %)	0 (0 %)	NA	NA	
	**rs2228000**	**Ala499Val**	**CC**	**443 (58.8** **%)**	**548 (42.6** **%)**	–	–	0.094
			**CT**	**269 (35.8** **%)**	**563 (43.7** **%)**	**<0.0001**	**0.59 (0.49**–**0.72)**	
			**TT**	**41 (5.4** **%)**	**177 (13.7** **%)**	**<0.0001**	**0.29 (0.20**–**0.41)**	
	rs2228001	Gln939Lys	AA	187 (39.7 %)	480 (36 %)	–	–	0.71
			AC	202 (42.9 %)	647 (48.4 %)	0.0616	0.80 (0.63–1.01)	
			CC	82 (17.4 %)	209 (15.6 %)	0.9378	0.99 (0.73–1.34)	
	rs3731062	Leu48Phe	CC	658 (96.6 %)	1,484 (94 %)	–	–	0.71
			CT	23 (3.4 %)	93 (5.9 %)	0.0100 (0.15*)	0.54 (0.33–0.86)	
			TT	0 (0 %)	1 (0.1 %)	NA	NA	
XPD	**rs1799793**	**Asp312Asn**	GG	219 (34.8 %)	573 (50.2 %)	–	–	0.94
			AG	235 (37.4 %)	541 (37.7 %)	0.2507	1.14 (0.91–1.41)	
			**AA**	**175 (27.8)**	**128 (12.1** **%)**	**<0.0001**	**3.58 (2.71**–**4.71)**	
	rs238406	Exon/intron	CC	231 (30.7 %)	356 (28.7 %)	–	–	<0.001
			AC	363 (48.1 %)	672 (54.3 %)	0.0826	0.83 (0.67–1.02)	
			AA	160 (21.2 %)	211 (17 %)	0.2408	1.17 (0.90–1.53)	
	rs18131	Lys751Gln	AA	244 (35.8 %)	592 (36.2 %)	–	–	0.30
			AC	327 (48 %)	767 (46.9 %)	0.7372	1.03 (0.85–1.26)	
			CC	110 (16.2 %)	276 (16.9 %)	0.8050	0.97 (0.74–1.26)	
XPE	rs1050244	UTR-3	CC	411 (86.7 %)	1,190 (87 %)	–	–	0.12
			CT	63 (13.4 %)	167 (12.2 %)	0.5570	1.10 (0.80–1.50)	
			TT	0 (0 %)	10 (0.7 %)	NA	NA	
XPF	rs762521	Exon/intron	GG	385 (51.2 %)	741 (58.7 %)	–	–	0.13
			AG	318 (42.3 %)	440 (34.8 %)	0.0005 (0.0075*)	1.40 (1.16–1.69)	
			AA	49 (6.5 %)	82 (6.5 %)	0.4240	1.17 (0.80–1.70)	
XPG	rs1047768	Exon/intron	CC	138 (29.8 %)	465 (35 %)	–	–	0.19
			CT	221 (47.7 %)	623 (46.8 %)	0.1272	1.21 (0.95–1.55)	
			TT	104 (22.5 %)	242 (18.2 %)	0.0124	1.47 (1.09–1.99)	
	rs1047769	Met254Val	AA	437 (95 %)	1,259 (92.8 %)	–	–	0.88
			AG	23 (5 %)	95 (7 %)	0.1392	0.70 (0.44–1.12)	
			GG	0 (0 %)	2 (0.2 %)	NA	NA	
	rs17655	Asp1104His	GG	429 (58.5 %)	869 (64 %)	–	–	<0.001
			CG	272 (37.1 %)	404 (29.7 %)	0.0018 (0.027*)	1.36 (1.12–1.65)	
			CC	32 (4.4 %)	85 (6.3 %)	0.2088	0.76 (0.50–1.16)	
	rs2227869	Cys529Ser	GG	372 (86.7 %)	1,168 (87.6 %)	–	–	0.13
			CG	55 (12.8 %)	162 (12.2 %)	0.6549	1.08 (0.78–1.50)	
			CC	2 (0.5 %)	2 (0.2 %)	0.2489	3.17 (0.45–22.61)	
	rs4150360	Exon/intron	TT	185 (25 %)	167 (25.8 %)	–	–	0.24
			CT	345 (46.5 %)	339 (52.2 %)	0.5299	0.92 (0.71–1.19)	
			CC	212 (28.5 %)	143 (22 %)	0.0546	1.34 (0.99–1.81)	

Bolded data indicate that these SNPs are significantly associated with an altered cancer risk

The prevalence of SNPs variants among CRC patients and controls

* p value after Bonferroni correction

#### Genotyping

Genotyping was performed using DNA isolated from peripheral blood samples taken from colorectal cancer patients and healthy controls according to the method of Miller et al. (1986) [26]. Molecular analysis was performed using a combination of real-time PCR (LightCycler 480, Roche, Penzberg, Germany) and MassARRAY MALDI-TOF MS analysis (Sequenom Inc., San Diego, CA, USA). For real-time PCR TaqMan probes were used (Applied Biosystems, Foster City, CA). MALDI-TOF analysis was based on a primer extension reaction to detect and determine the SNP allele. Reactions were performed according to the manufacturer’s instructions.

#### Sequencing

Random DNA samples were sequenced to verify the results of the MassARRAY genotyping and real-time PCR analysis (data not shown). Sequencing was conducted using universal primers in combination with the ABI PRISM BigDye Terminator Cycle kit (Applied Biosystems, Foster City, CA). The sequencing was performed in the 3130 Genetic Analyzer (Applied Biosystems, Foster City, CA).

#### Statistics

Possible deviation of the allele frequencies from those expected under Hardy–Weinberg equilibrium (HWE) was also assessed using the Chi square analysis [27]. Genotype frequency differences, the odds ratio (OR) and 95 % confidence intervals (95 % Cl) were estimated using regression analysis for additive model of inheritance. Logistic regression analysis was performed using R software environment version 2.15.0 [28].

Haplotype frequencies and their potential association with disease risk were estimated using the haplo.stats CRAN package (version 1.5.5) by Sinnwell and Schaid for an R environment [29].

Linkage disequilibrium between SNPs for a given haplotype was calculated using the software JLIN by Carter et al. [30] as described in a previous study [24].

Bonferroni correction for multiple testing was applied to all results that demonstrated a significant difference between the cases and controls.

## Results

All SNPs tested were in Hardy–Weinberg Equilibrium within the control and CRC populations under study. This indicates that any differences observed in the study population are a result of differences attributable to those SNPs that are significantly different in frequency between the control and the colorectal cancer population.

A number of SNPs were found to be in linkage disequilibrium (LD). These were in *XPC*: rs2228001, G1475A, G2061A, rs2228000 and rs3731062 that we have previously observed [24].

From the 15 SNPs analyzed, logistic regression revealed one SNP in *XPC* and one in *XPD* was associated with colorectal cancer risk. A significantly decreased colorectal cancer risk was identified for the *XPC* rs2228000 (Ala499Val) CT genotype (OR 0.59; p < 0.0001) and for TT genotype (OR 0.29; p < 0.0001) compared to the reference genotype (CC). And an increased risk of disease was associated with the *XPD* SNP, rs1799793 (Asp312Asn-) AG genotype (OR 1.44; p = 0.018) and for AA genotype (OR 3.31; p < 0.0001) compared to the control population. These results all remained significant after Bonferroni Correction (see Table 1). None of the other SNPs were found to be associated with colorectal cancer risk.

### Gender and colorectal cancer risk

#### *XPC* gene

Gender stratification analysis revealed differences in the association of the rs2228000_TT genotype in females compared to males (Table 2). Women who were heterozygous for this SNP appeared to have a significantly reduced risk of colorectal cancer compared to the control population (OR 0.2; 95 % CI 0.15–0.28) which was further reduced in the homozygous state (OR 0.1; 95 % CI = 0.06–0.17) whereas in males there was no association in the heterozygous state but a similar reduced risk if homozygous (OR 0.44; 95 % CI 0.26–0.75).

**Table 2:** Stratified analysis by gender

Gene	SNP	Genotype	Females		Males	
			p value	OR (95 % Cl)	p value	OR (95 % Cl)
XPC	G2061A	GG	–	–	–	–
		AG	0.6266	1.09 (0.77–1.54)	0.0128 (0.192*)	0.66 (0.47–0.91)
		AA	0.7988	1.09 (0.57–2.10)	0.4657	0.77 (0.38–1.56)
	G1475A	GG	–	–	–	–
		AG	0.5222	1.19 (0.70–2.02)	0.5362	0.84 (0.49–1.45)
		AA	0.9927	NA	NA	NA
	**rs2228000**	CC	**–**	**–**	**–**	–
		**CT**	**<0.0001**	**0.20 (0.15**–**0.28)**	0.5088	0.92 (0.71–1.19)
		**TT**	**<0.0001**	**0.10 (0.06**–**0.17)**	0.0022 (0.033*)	**0.44 (0.26**–**0.75)**
	rs2228001	AA	–	–	–	–
		AC	0.1939	0.80 (0.57–1.12)	0.2331	0.82 (0.60–1.13)
		CC	0.3301	0.79 (0.49–1.27)	0.3917	1.20 (0.79–1.80)
	rs3731062	CC	–	–	–	–
		CT	0.0808	0.48 (0.21–1.09)	0.0622	0.58 (0.32–1.03)
		TT	NA	NA	0.9953	NA
XPD	**rs1799793**	GG	**–**	**–**	**–**	–
		**AG**	0.7960	1.04 (0.76–1.43)	**<0.0001**	**1.92 (1.41**–**2.62)**
		**AA**	0.0070 (0.105*)	1.70 (1.16–2.49)	**<0.0001**	**6.92 (4.61**–**10.36)**
	rs18131	AA	–	–	–	–
		AC	0.1379	1.25 (0.93–1.68)	0.3792	0.89 (0.68–1.16)
		CC	0.1208	1.36 (0.92–2.00)	0.0802	0.72 (0.49–1.04)
XPE	rs1050244	CC	–	–	–	–
		CT	0.2975	0.77 (0.47–1.26)	0.0825	1.43 (0.96–2.14)
		TT	0.9947	NA	0.9939	NA
XPF	rs762521	GG	–	–	–	–
		AG	<0.0001	1.87 (1.40–2.50)	0.3114	1.14 (0.88–1.47)
		AA	0.3005	1.35 (0.76–2.39)	0.8596	1.05 (0.63–1.73)
XPG	rs1047768	CC	–	–	–	–
		CT	0.0862	1.37 (0.96–1.97)	0.5973	1.09 (0.78–1.53)
		TT	0.0209 (0.314*)	1.71 (1.08–2.69)	0.1614	1.33 (0.89–1.99)
	rs1047769	AA	–	–	–	–
		AG	0.2322	0.67 (0.35–1.29)	0.3320	0.72 (0.37–1.40)
		GG	NA	NA	0.9940	NA
	rs2227869	GG	–	–	–	–
		CG	0.5842	1.15 (0.70–1.87)	0.8788	1.04 (0.66–1.61)
		CC	0.4710	2.78 (0.17–44.64)	0.3729	3.53 (0.22–56.76)
	rs4150360	TT	–	–	–	–
		CT	0.3853	0.85 (0.61–1.21)	0.5580	0.88 (0.58–1.34)
		CC	0.0696	1.44 (0.97–2.13)	0.6981	1.10 (0.68–1.77)

Bolded data indicate that these SNPs are significantly associated with an altered cancer risk

Odds ratio (95 % CI) and p value for females and males from polish population for all examined SNPs

* p value after Bonferroni correction

#### *XPD* gene

The polymorphisms in *XPD* (rs1799793, rs238406) were both associated with colorectal cancer development. Both AA and AG genotypes in *XPD* rs1799793 were strongly associated with increasing cancer risk in males (OR 6.92, 95 % CI 4.61–10.36; OR 1.92, 95 % CI 1.42–2.62), whereas in females there appeared to be an association with homozygosity but no relationship with heterozygosity. After Bonferroni correction, the association with males and risk remained but became non-significant in females. For *XPD* rs238406_AA was significantly associated with cancer development in women (OR 2.02, p = 0.006) but not in men. The opposite tendency was observed in male CRC patients, where the *XPD* rs238406_AA and AC genotypes were associated with a decreased risk of disease (OR 0.69, CI 0.47–0.99; and OR 0.51, CI 0.38–0.68, respectively). After Bonferroni correction only males that were heterozygous for the rs238406 SNP were associated with a decrease in cancer risk.

### Age and colorectal cancer risk

Dichotomizing the patients into two groups (those under 50 years of age and those equal to or above 50 years of age) revealed an association of the 2 SNPs within *XPC* and *XPD* genes with colorectal cancer risk (Table 3).

**Table 3:** Stratified analysis by age

Gene	SNP	Genotype	<50		>50	
			p value	OR (95 % Cl)	p value	OR (95 % Cl)
XPC	G2061A	GG	–	–	–	–
		AG	0.6592	0.88 (0.49–1.57)	0.0921	0.80 (0.61–1.04)
		AA	0.2556	0.31 (0.04–2.35)	0.9347	0.98 (0.59–1.62)
	G1475A	GG	–	–	–	–
		AG	0.6344	1.23 (0.51–2.95)	0.7875	0.94 (0.62–1.43)
		AA	NA	NA	0.9927	NA
	**rs2228000**	CC	**–**	**–**	**–**	–
		**CT**	0.1300	0.70 (0.45–1.11)	**<0.0001**	**0.59 (0.47**–**0.73)**
		**TT**	0.0608	0.46 (0.20–1.04)	**<0.0001**	**0.27 (0.18**–**0.40)**
	rs2228001	AA	–	–	–	–
		AC	0.3833	1.29 (0.73–2.26)	0.0146 (0.2190*)	0.73 (0.56–0.94)
		CC	0.2964	1.48 (0.71–3.08)	0.5922	0.91 (0.65–1.28)
	rs3731062	CC	–	–	–	–
		CT	0.2294	0.4739 (0.14–1.60)	0.0240 (0.3600*)	0.55 (0.33–0.92)
		TT	NA	NA	0.9950	NA
XPD	**rs1799793**	GG	**–**	**–**	**–**	–
		**AG**	0.4295	1.25 (0.72–2.15)	0.0033 (0.0495*)	**1.44 (1.13**–**1.83)**
		**AA**	**<0.0001**	**3.64 (2.01**–**6.61)**	**<0.0001**	**3.23 (2.38**–**4.38)**
	rs18131	AA	–	–	–	–
		AC	0.1587	1.40 (0.88–2.24)	0.5688	0.94 (0.75–1.17)
		CC	0.6260	1.17 (0.62–2.22)	0.4989	0.90 (0.67–1.21)
XPE	rs1050244	CC	–	–	–	–
		CT	0.9110	1.04 (0.50–2.17)	0.4748	1.14 (0.80–1.60)
		TT	0.9939	NA	0.9949	NA
XPF	rs762521	GG	–	–	–	–
		AG	0.1853	1.35 (0.87–2.12)	0.0015 (0.0225*)	1.41 (1.14–1.75)
		AA	0.5595	1.29 (0.54–3.07)	0.5523	1.14 (0.75–1.73)
XPG	rs1047768	CC	–	–	–	–
		CT	0.6051	1.16 (0.66–2.04)	0.1669	1.21 (0.92–1.60)
		TT	0.6505	0.83 (0.36–1.89)	0.0089 (0.1335*)	1.55 (1.12–2.15)
	rs1047769	AA	–	–	–	–
		AG	0.7372	1.19 (0.43–3.25)	0.0697	0.61 (0.36–1.04)
		GG	NA	NA	0.9935	NA
	rs2227869	GG	–	–	–	–
		CG	0.9470	1.03 (0.48–2.21)	0.5743	1.11 (0.77–1.60)
		CC	0.9947	NA	0.1643	5.50 (0.50–60.92)
	rs4150360	TT	–	–	–	–
		CT	0.5185	0.83 (0.48–1.45)	0.7153	0.95 (0.70–1.27)
		CC	0.3042	0.70 (0.35–1.39)	0.0177 (0.2655*)	1.51 (1.07–2.12)

Bolded data indicate that these SNPs are significantly associated with an altered cancer risk

Odds ratio (95 % CI) and p value for patients diagnosed under 50 and over 50 years old for all examined SNPs

* p value after Bonferroni correction

Both *XPC* rs2228000_TT and CT genotypes decreased cancer risk only in patients over 50 years of age (OR 0.27 CI 0.18–0.40; OR 0.59 CI 0.47–0.73, respectively). The significance remained after Bonferroni correction.

For *XPD* rs1799793 disease risk did not appear to differ between the two age groups for patients homozygous for the SNP, for patients <50 years of age OR 3.64 CI 2.01–6.61) and patients ≥50 years of age (OR 3.23 CI 3.23–4.38) among patients having homozygotes variant AA at XPD rs1799793. Patients who were heterozygous for this SNP, there appeared to be a tendency, which was in the same direction of the causal SNP, of an increased risk of disease. For patients over 50 years of age, there was a statistically significant increase in disease risk although the effect size was somewhat modest. For patients under 50 years of age, a tendency towards an association was observed but it was not statistically significant.

### Haplotype analysis

#### XPC

Using the Haplo.stats CRAN package (version 1.5.5) [29] we undertook haplotype analysis for *XPC.* The analysis revealed three haplotypes that were associated with a significantly decreased disease risk and one that significantly increased colorectal cancer risk compared to the reference haplotype: GCGCC (see Table 4). Moreover, the haplotype GAGCC appeared to be associated with a significant increase in disease risk (OR 2.56 CI 1.94–3.39). For the remaining haplotypes, no significant differences in disease risk between cases and controls were identified (Table 4).

**Table 4:** Haplotype frequency of examined XPC, XPD, XPG variants between cases and controls

Gene	SNPs					p	OR	95 % Cl
XPC	G1475A	rs2228001	G2061A	rs2228000	rs3731062			
	G	C	G	C	C	Ref.	Ref.	Ref.
	**G**	**A**	**G**	**T**	**C**	**<0.0001**	**0.43**	**(0.31**–**0.60)**
	**G**	**A**	**G**	**T**	**T**	**<0.0001**	**0.09**	**(0.02**–**0.41)**
	**A**	**A**	**G**	**T**	**C**	**<0.0001**	**0.56**	**(0.32**–**0.99)**
	G	A	A	T	C	0.5282	1.71	(0.05–2.89)
	G	C	G	T	C	0.9878	1.46	(1.00–2.14)
	G	A	A	C	C	0.1475	1.04	(0.81–1.33)
	**G**	**A**	**G**	**C**	**C**	**<0.0001**	**2.56**	**(1.94**–**3.39)**
XPD	2253	RS1799793						
	A	G	–	**–**	**–**	Ref.	Ref.	Ref.
	C	G	–	–	–	<0.0001	0.99	(0.79–1.25)
	**C**	**A**	**–**	**–**	**–**	**0.005**	**1.33**	**(1.14**–**1.54)**
	**A**	**A**	**–**	**–**	**–**	**<0.0001**	**3.20**	**(2.50**–**4.09)**
XPG	rs1047768	rs1047769	rs2227869	rs4150360				
	C	A	G	T	–	Ref.	Ref.	Ref.
	C	G	G	T	–	0.053	0.74	(0.38–1.41)
	C	A	C	C	–	0.785	1.14	(0.77–1.68)
	T	A	G	C	–	0.526	1.21	(1.03–1.42)
	C	A	C	T	–	0.066	6.67	(0.46–96.86)
	**C**	**A**	**G**	**C**	**–**	**0.002**	**3.82**	**(2.25**–**6.46)**
	**T**	**A**	**G**	**T**	**–**	**<0.0001**	**4.38**	**(2.51**–**7.66)**

Bolded data indicate that these SNPs are significantly associated with an altered cancer risk

Haplotypes not frequent enough to allow haplotype analysis were excluded

#### XPD

Haplotype analysis of *XPD* revealed two protective haplotypes and three that were associated with an increase in disease risk (Table 4).

#### XPG

Differences in the haplotype structure revealed two that were protective against colorectal cancer and two that were linked to an enhanced risk of disease (Table 4).

## Discussion

In this case–control study that included 758 patients with colorectal cancer and 1,841 healthy adults, we found that the *XPC* rs2228000 (Ala499Val) and the *XPD* rs1799793 (Asp312Asn-) polymorphisms were significantly associated with an altered colorectal cancer risk.

Logistic regression revealed that the *XPC* rs2228000_CT or _TT genotype was significantly associated with a decreased colorectal cancer risk. Gender stratification demonstrated that this correlation is more evident among women than men. Haplotype analysis of the XPC gene suggested that there are 3 protective *XPC* alleles. We found no association between the remaining variants of XPC and colorectal cancer risk.

To the best of our knowledge, only a few studies have investigated the role of *XPC* in colorectal cancer and the published data remains inconclusive. None of these studies demonstrated a clear association rs2228000 polymorphisms with colorectal cancer risk. Huang et al. examined 772 patients with distal colon cancer and 777 controls and suggested no correlation between all examined *XPC* polymorphisms (Arg492His, Ala499Val, Lys939Gln) and colorectal cancer risk, however they suggested that the smoking-associated risk in CRC was modified by haplotypes containing the *XPC* 499Val variant [21]. Wu et al. assessed the correlation between *XPC* polymorphisms and colorectal cancer development among 421 CRC patients and 845 controls. The study showed no significant association between Ala499Val and CRC risk, but a significant decrease risk of rectal cancer correlated with CT and TT genotypes [12]. Berndt et al. examined 5 SNPs in XPC among 250 colorectal cancer patients and 2,228 controls, authors suggested increased risk of colorectal cancer among patients having at least one 492His allele of the XPC Arg492His (rs2227999) and no association of the remaining SNPs (including rs2228001 and rs2228000) with colorectal cancer [13].

A meta-analysis focused on the role of *XPC* in carcinogenesis, which revealed two SNPs in *XPC* (rs2228001 and rs2228000) that were significantly associated with overall cancer risk. A meta-analysis that included 6 studies examined *XPC* rs2228001 as a risk factor for colorectal cancer (2,751 cases and 3,607 controls) but failed to include studies examining the role of *XPC* rs2228000. This report did, however, show that rs2228001 was associated with colorectal cancer risk [14].

Similar conclusions have been drawn from reports from Liu et al. [11] and Wu, et al. (2011) [12]. In Liu et al., after evaluation of 1028 CRC cases and 1,085 controls they found that the Lys939Gln (AC or CC genotype) was associated with an increased risk of colorectal cancer [11]. In the other study, which evaluated 421 colorectal cancer patients and 845 healthy individuals the results suggested that the CC genotype in the Lys939Gln and PAT +/+ genotype in an intronic biallelic poly(AT) insertion/deletion polymorphism in *XPC* increased CRC risk [14]. Our results of *XPC* suggested no association between Lys939Gln with colorectal cancer risk and confirmed the conclusions from two previous reports [18, 23].

Logistic regression analysis of *XPD* genotypes in our study revealed an association with *XPD* rs1799793 (Asp312Asn-) AG and AA genotypes with an increased colorectal cancer risk. This association appeared greater in men. Previously published data focusing on the correlation between *XPD* gene polymorphisms and the risk of colorectal cancer development were inconclusive. Some reports have suggested an association of *XPD* polymorphisms with CRC [15, 16, 32] whereas others have not observed any such correlation [13, 16, 19, 21, 23, 31].

There have been eight previous reports examining relationship between Asp312Asn polymorphism and CRC and most have failed to detect any association [13, 16, 19, 21, 23, 31]. Only Jelonek et al. examined 153 CRC cases and 507 controls from the Polish population and found that the AA genotype in *XPD* Asp312Asn- was overrepresented in the colorectal cancer group [32].

Most of the reports are concentrated on assessing the impact of Lys751Gln polymorphism on CRC risk. The results from the present study revealed a lack of association between X*PD* Lys751Gln and colorectal cancer risk, which confirmed several previous reports [17, 18, 33]. Likewise, another Polish report utilizing 100 cases and 100 controls suggested that the *XPD* Lys751Gln did not appear to contribute significantly to disease risk in the Polish population [34]. In contrast to our findings, Jelonek et al. [32] found that *XPD* 751Gln (C allele) was more frequent in the cancer group but failed to reach statistical significance. Whereas Skjelbred et al. suggested that carriers of the variant genotype of XPD Lys751Gln had an increased risk of low-risk adenomas but not carcinomas [15]. Another Polish study (100 CRC cases and 100 controls) reported that XPD Lys751Gln was associated with decreased risk of CRC for individuals harboring at least one A allele [16]. All these studies were performed on relatively small samples size (less than 200 cases) and each suffered from a lack of power to detect small effect sizes.

A meta-analysis that included 15 studies (Lys751Gln) containing 3,042 CRC cases and 4,672 controls and seven studies (Asp312Asn) containing 1,581 cases and 2,846 controls, suggested that both polymorphisms in *XPD* are not significantly associated with CRC risk [19]. Similar findings were obtained by Stern et al. [20], Huang et al. [21], Moreno, et al. [22] and Hansen et al. [23]. In these studies the groups examined consisted of, 310 cases and 1,176 controls [20], 772 cases and 777 controls [21], 377 cases and 329 controls [22], 405 cases and 810 controls [23]. All reports failed to identify any correlation between these SNPs and the risk of colorectal cancer. Our observation confirm previous findings with respect to the *XPD* Lys751Gln polymorphism demonstrating it is not associated with colorectal cancer risk. We could not, however, explain the negative results of XPD Asp312Asn. This discrepancy and conflicting data may be a result of population stratification or the relatively small numbers of cases used in the study.

All SNPs in *XPE, XPF, XPG* that were examined in this study, did not appear to contribute significantly to disease risk in the Polish population, except for *XPG* rs17655 (Asp1104His). The heterozygous genotype CG of rs17655 was associated with an increased risk of colorectal cancer, which confirmed the findings of Liu et al. [11]. The analysis undertaken by Liu et al. [11] was performed on 1,557 CRC patients and 1,085 controls. Our results confirmed previous reports about the lack of any association between *XPE, XPF, XPG* polymorphisms with colorectal cancer risk [13, 16, 21, 22, 31, 35].

To our knowledge, there is little if any haplotype data available for the assessment of disease risk between NER genes and colorectal cancer. In this study we have examined several haplotypes in *XPC, XPD* and *XPG*. Many of the SNPs examined result in amino acid substitutions, which have the potential to alter the kinetic properties of the respective NER protein. When investigating XPG, no individual SNP was found to be associated with any change in CRC risk but we observed four haplotypes that were significant, two that increased risk and two that decreased risk. This provides some evidence to suggest that combinations of polymorphisms should be examined in more detail to reveal associations that are not immediately apparent.

In conclusion, this study suggests that *XPC* rs2228000 (Ala499Val) and the *XPD* rs1799793 (Asp312Asn-) polymorphisms are significantly associated with an alteration in colorectal cancer risk. However, it remains necessary to conduct additional larger studies taking into account environmental factors to assess gene environment interactions to fully understand the relationship between NER and colorectal cancer susceptibility. Future studies will result in a better understanding of the role of NER gene polymorphisms in colorectal cancer development.
